# Effect of Vitamin D3 on Transected and Crushed Injuries in Rat Sciatic Nerve Healing

**DOI:** 10.3390/biomedicines14020481

**Published:** 2026-02-22

**Authors:** Inanc Dogan Cicek, Handan Derebasinlioglu, Ayse Demirkazik, Hatice Reyhan Egilmez

**Affiliations:** 1 Department of Plastic Reconstructive and Aesthetic Surgery, Faculty of Medicine, Cumhuriyet University, 58140 Sivas, Türkiye; 2 Department of Plastic Reconstructive and Aesthetic Surgery, Faculty of Medicine, Inonu University, 44280 Malatya, Türkiye; 3 Department of Biophysics, Faculty of Medicine, Cumhuriyet University, 58140 Sivas, Türkiye; 4 Department of Pathology, Faculty of Medicine, Cumhuriyet University, 58140 Sivas, Türkiye

**Keywords:** peripheral nerve injury, vitamin D3, action potential, hot plate test

## Abstract

**Background:** Peripheral nerve injury can happen for a variety of causes. Despite major breakthroughs in microsurgery, nerve repair results are not always sufficient. **Methods:** Thirty-two Wistar albino rats were split into four groups: primary nerve repair (PNR), PNR with vitamin D3 treatment, nerve crush injury (NCI), and NCI with vitamin D3 treatment. In the PNR + D3 and NCI + D3 groups, 1 mcg/kg of vitamin D3 was given intraperitoneally on days 1, 3, 5, and 7 of the 12-week healing period. Electrophysiological measurements were taken prior to the injury. At 12 weeks after damage, a hot plate test was performed to assess acute pain, and the electrophysiological measurements were repeated. Before the rats were sacrificed, biopsy samples from the right sciatic nerve were collected for histopathological evaluation. **Results:** Post-healing action potential values were not statistically different between the PNR and PNR + D3 groups; however, they were considerably lower in the NCI + D3 group than in the NCI group. The reaction time in the hot plate test was considerably slower in the D3-treated groups compared to the control groups. Histopathology score was substantially higher in the PNR + D3 group as compared to the PNR group, and lower in the NCI + D3 group as compared to the NCI group. **Conclusions:** Other than improved myelination, vitamin D3 treatment following primary repair of transected nerves produced no statistically significant improvement. Vitamin D3 treatment caused a negative impact on the crush injury, as assessed by the findings of histopathology and electrophysiological measurements. Overall, the results indicate that the efficacy of vitamin D3 treatment may vary depending on the type of injury.

## 1. Introduction

The peripheral nervous system allows a person to interact with and adapt to the outside world. Problems with this system have a detrimental impact on quality of life and can result in psychological difficulties. The most prevalent cause of peripheral nerve damage is trauma [1]. Following an accident, recovery may be partial, and aberrant healing might result in functional loss and chronic pain. Treatment planning for such injuries is critical to achieving functional recovery. As a result, the treatment is centered on microsurgical procedures that accomplish tension-free repairs of the severed nerve terminals while maintaining normal fascicular alignment [2]. Although there are several different classifications of peripheral nerve injuries, they can be broadly classified into two categories: transection and crush injuries. They both have distinct features influencing the pathophysiology and the repair process [3]. In transection injuries, complete disruption of axons and connective tissue sheaths takes place; therefore, surgical treatment is the only option to allow repair to take place [4]. In crush injuries, endoneurial tubes and basal lamina are mostly preserved, allowing regenerating axons to follow their original pathways, which generally results in faster and more predictable functional recovery [5]. Therefore, these pathophysiological differences can have an effect on the choice of different treatment strategies.

Significant breakthroughs in treatment regimens have been made as we gain a better understanding of the pathophysiology of peripheral nervous system damage and regeneration, as well as the increased utilization of microsurgical procedures. In spite of these developments, adequate and expected nerve repair does not always take place. Therefore, new therapeutic strategies for better regenerative treatments are still needed, and a variety of chemicals are currently under investigation for their effects on nerve healing [6,7,8,9,10].

Besides its well-known effects on musculoskeletal health, vitamin D has also been studied as a treatment strategy for a variety of extraskeletal conditions, including central nervous system traumas, diabetes mellitus, amyotrophic lateral sclerosis, multiple sclerosis, and metabolic syndrome [11,12,13]. In the nervous system, vitamin D3 has been shown to exert some beneficial effects in demyelination models in mice [14,15] and diabetic peripheral neuropathies in humans [16,17]. Vitamin D3 has also been indicated to play roles in age-related neurodegenerative diseases such as Alzheimer’s disease, Parkinson’s disease, and multiple sclerosis, and its deficiency has been associated with cognitive decline [18]. However, the effects of vitamin D3 in humans are not conclusive, and the clinical evidence of true nerve regeneration or complete functional restoration remains limited and inconsistent [19].

Few investigations have indicated that vitamin D might promote peripheral nerve repair in experimental rat models [20,21,22]. As a treatment regimen, vitamin D was given orally in regular doses in these studies. However, the application of daily oral doses may not usually be a viable strategy. To increase the bioavailability of an agent, injectable preparations may be required to provide more consistent results. Therefore, the aim of this study is to investigate the effect of intraperitoneal vitamin D3 treatment on the peripheral nerve transection (neurotmesis) and crush injuries.

## 2. Materials and Methods

### 2.1. Experimental Design

Ethical approval of the study was provided by the Institutional Animal Ethics Committee of Cumhuriyet University (Approval code: 187; Approval date: 17 January 2019). The study included a total of 32 Wistar albino rats weighing 230–300 g. All animals were housed in a temperature and humidity-controlled environment for a period of two weeks for acclimatization before the study. Anesthesia was achieved with an intraperitoneal injection of ketamine (90 mg/kg) and xylazine (3 mg/kg). Following general anesthesia, a skin incision was performed around the right hip joint fold. The biceps femoris muscle was found and incised to reveal the sciatic nerve. Electrophysiological measures were taken from all rats’ right sciatic nerves prior to inducing experimental injury.

Rats were first equally divided into two groups as the primary nerve repair (PNR) and the nerve crush injury (NCI) group. Using a clean scalpel, 16 rats underwent right sciatic nerve neurotmesis 1 cm distal to the sciatic notch. The transected sciatic nerves were all restored end-to-end by the same surgeon under a microscope (ZEISS, OPMI VARIO 700, Jena, Germany) with interrupted sutures of 8-0 round monofilament polypropylene. In the remaining 16 rats, a crush injury was induced by clamping the right sciatic nerve 1 cm distal to the sciatic notch for 1 min using a spring-loaded microvascular bulldog clamp [23]. To ensure uniformity, all applications were performed by the same person using the same clamp. The rats in each group were further separated into two groups, each containing 8 rats. Vitamin D3 dosage was chosen based on a previous study [24]. The final groups and treatments were as follows:

PNR group: Following sciatic nerve transection and repair, the rats received no further treatment during the 12-week recovery period.

PNR + D3 group: Following sciatic nerve transection and repair, the rats were given 1 mcg/kg of vitamin D3 intraperitoneally on days 1, 3, 5, and 7 of the 12-week healing period.

NCI group: Following sciatic nerve crush injury, the rats had no additional operations throughout the 12-week recovery period.

NCI + D3 group: Following sciatic nerve crush damage, the rats were given 1 mcg/kg of vitamin D3 intraperitoneally on days 1, 3, 5, and 7 of the 12-week recovery period.

At 12 weeks, all rats underwent a hot plate test for functional evaluation, and electrophysiological recordings of the injured sciatic nerves were repeated. Upon completion of the hot plate tests, all animals were sacrificed under xylene-ketamine anesthesia by cervical decapitation. After the euthanasia, right sciatic nerve samples were collected and preserved in 10% formaldehyde for histopathological and immunohistochemical evaluation.

### 2.2. Electrophysiological Assessment

The rat sciatic nerves were examined for action potential (∆PP) parameters such as amplitude (mV), depolarization time (∆DT, ms), repolarization time (∆RT, ms), and total activation time (∆DT + ∆RT, ms). Baseline values were determined using AP parameters before the injury and compared to the values obtained after the 12-week recovery period. Measurements were carried out using an ADInstruments (Sydney, Australia) Animal Bio amplifier and stimulator with a platinum probe. Analyses were carried out using the ADInstruments LabChart 7 program.

### 2.3. Hot Plate Test

Hot plate test was repeated for each rat before and after 12 weeks of experimental damage. The rats were placed on a hot plate (AHP May 0603 Hot Plate Analgesic Commat, Ankara, Turkey) adjusted to 55 ± 0.5 °C. To avoid paw injury, the rats were removed from the hot plate if they did not respond within the 30 s time limit. Following a 5 min recoup period, hot plate measurements were taken at three intervals. The time from placement on the hot plate to the first hind paw lick or jump was measured with a stopwatch, and the median was determined. Hot plate test findings were calculated as the percentage of maximum probable effect (%MPE) = [(test time − basal latency time)/cut-off time − basal latency time] × 100.

### 2.4. Histopathological and Immunohistochemical Evaluation

Sciatic nerve samples were fixed in 10% formaldehyde and then embedded in paraffin. Using a microtome, 2.5 µm thick sections were cut from the paraffin blocks and placed on positively charged slides. The slides were routinely stained with hematoxylin and eosin (H&E) and Masson’s trichrome stains. An avidin-biotin peroxidase-based immunohistochemistry was also performed for neurofilament (SantaCruz, Dallas, TX, USA, clone: 4E10-D8-F4 diluted 1:100) and S-100 (SantaCruz, Dallas, TX, USA, clone: S1-61 diluted 1:100) with diaminobenzidine/H_2_O_2_ reaction. All slides were blindly examined and evaluated by the same pathologist. Neurofilament immunoreactivity was used to assess axonal regeneration, while S-100 was used to show Schwann cells in the tissue sections. In histopathologic examinations, the evaluation criteria included the presence or absence of nodulation, foam cells, and collagen deposition. Immunoreactivity of S-100 for Schwann cell proliferation and neurofilament for axonal regeneration was evaluated based on mild (<10%), moderate (10–50%), and severe (>50%) immunostained area in 5 different microscopic fields under 40x objective. The images obtained from the microscopic view were evaluated using Fiji (version 2.12.0) digital pathology image analysis software [25], and the ratios of immunostained fields were calculated.

### 2.5. Statistical Analysis

Based on previous experimental studies in the literature [23], sample size estimation indicated that 8 animals per group would be sufficient to detect large differences in parameters with a significance level of 0.05 and a statistical power of 80%. The data from the study were analyzed with SPSS version 23.0 software (IBM Corp., Armonk, NY, USA). Normality was tested using the Shapiro–Wilk test (*p* > 0.05). The statistical comparisons of the ΔPP (mV) were performed by paired *t*-test (within-group) and independent-samples *t*-test (between-group). ΔDT, ΔRT, and ΔDT + ΔRT values were analyzed using the Wilcoxon signed-rank test (within-group) and Mann–Whitney U test (between-group). The statistical comparisons of hot plate test parameters were analyzed using the Kruskal–Wallis H test followed by post hoc Mann–Whitney U tests. The histopathological and immunohistochemical findings were compared using the chi-square test. Normally distributed parameters were presented as mean ± standard deviation (SD), whereas non-normally distributed data were presented as median (min–max), and *p* < 0.05 was considered statistically significant.

## 3. Results

### 3.1. Electrophysiological Findings

The results and the statistical comparison of the measurements of action potential within and between the groups are given in Table 1. There were no significant differences between the PNR and PNR + D3 groups in terms of pre-injury and post-healing ΔPP values (*p* > 0.05). After recovery, the NCI group showed significantly higher ΔPP values than the NCI + D3 group (*p* = 0.027).

Depolarization and repolarization times of the injured nerves were also measured. The PNR and PNR + D3 groups showed no significant change in post-healing ΔDT values (*p* > 0.05). After recovery, the ΔDT values were significantly higher in the NCI group than in the NCI + D3 group (*p* = 0.032). In both the PNR and PNR + D3 groups, ΔRT values increased significantly after healing compared with pre-injury values (*p* = 0.028). The NCI + D3 group showed a significant increase in ΔRT values after recovery compared with pre-injury levels (*p* = 0.017), whereas the change in the NCI group was not significant (*p* = 0.069).

Both the PNR and PNR + D3 groups had significantly increased post-healing ΔDT + ΔRT values compared with pre-injury levels (*p* = 0.025 and *p* = 0.028, respectively). The NCI group had significantly higher post-healing ΔDT + ΔRT values than the NCI + D3 group (*p* = 0.027). The within-group analysis also showed a significant increase in ΔDT + ΔRT values compared to the NCI group after recovery (*p* = 0.036). There was no significant change between pre-injury and post-healing ΔDT + ΔRT values in the NCI + D3 group (*p* = 0.208).

### 3.2. Hot Plate Test Findings

The median %MPE values between PNR and NCI groups, as well as between PNR + D3 and NCI + D3 groups, did not show any significance (*p* > 0.05). However, the median %MPE values were significantly higher in the vitamin D3-given groups as compared to the counterpart ungiven groups (*p* < 0.05). Median %MPE was higher in the PNR + D3 group than in the control, PNR, and NCI groups. Similarly, the NCI + D3 group had a greater median %MPE than the control, PNR, and NCI groups (Table 2).

### 3.3. Histopathological and Immunohistochemical Findings

**Figure 1:**
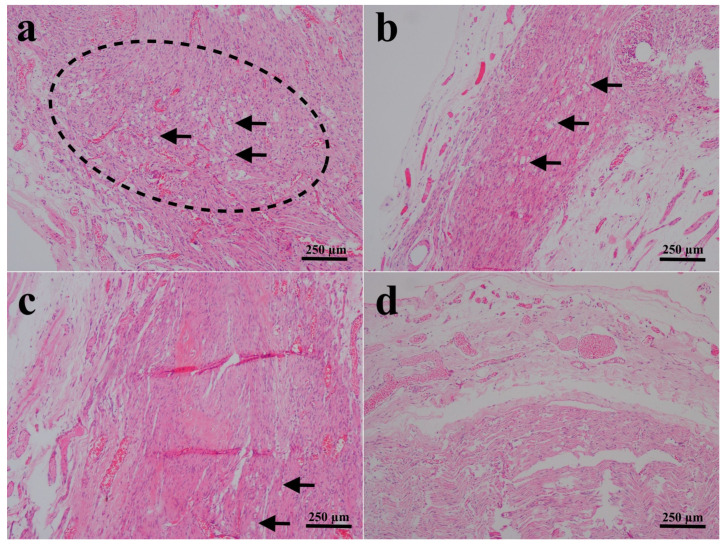
Histopathologic views of the sciatic nerve fibers. (**a**) PNR group: Irregularly arranged nerve fibers indicative of nodulation (dashed line) and prominent foam cells (arrows), (**b**) PNR + D3 group: Irregularly arranged nerve fibers and prominent foam cells (arrows), (**c**) NCI group: Regenerative nerve fibers with few foam cells (arrows), and (**d**) NCI + D3 groups: No nodulation and foam cells. H&E.

**Figure 2:**
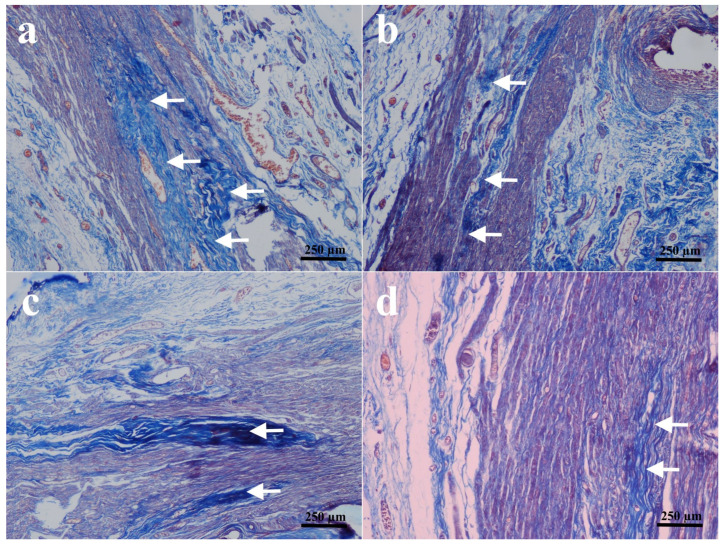
Histologic view of collagen deposition in the sciatic nerve fiber. (**a**) PNR group: Prominent collagen deposition (arrows) among the nerve fibers, (**b**) PNR + D3 group: Prominent collagen deposition (arrows) similar to PNR group, (**c**) NCI group: Moderate to heavy collagen deposition (arrows) among the regenerating nerve fibers, and (**d**) NCI + D3 groups: Less collagen deposition (arrows) compared to NCI group. Masson’s trichrome.

**Figure 3:**
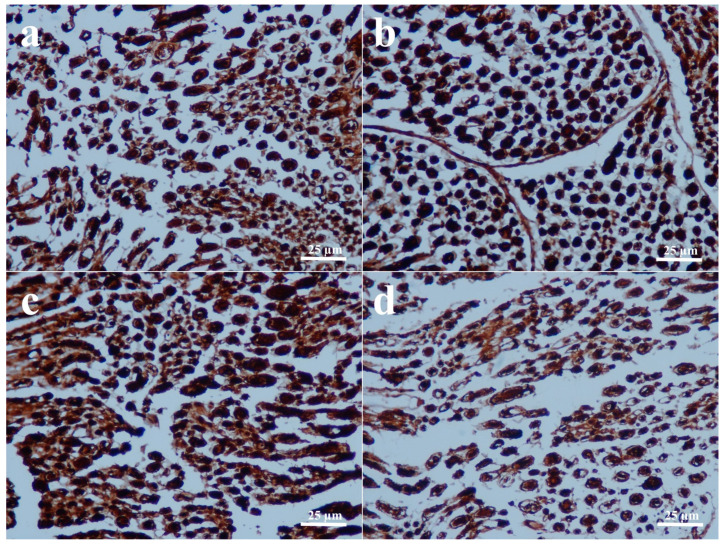
S-100 immunoreactivity as an indicator of Schwann cells. Less percentile of Schwann cells is present in the regenerating sciatic nerve fibers in the PNR group (**a**) compared to the PNR + D3 group (**b**), and a higher percentile of Schwann cells in the NCI group (**c**) compared to the NCI + D3 group (**d**).

**Figure 4:**
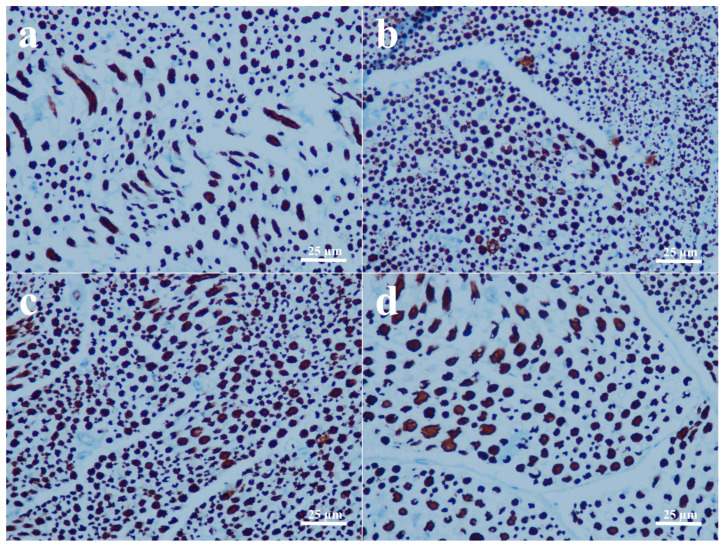
Neurofilament immunoreactivity as an indicator of axonal regeneration. The percentile of anti-neurofilament antibody immunoreactive axons is less in the PNR group (**a**) compared to the PNR + D3 group (**b**), and the percentile of regenerating axons is comparatively higher in the NCI group (**c**) compared to the NCI + D3 group (**d**).

Direct assessment of the sciatic nerves in the groups that received end-to-end primary nerve restoration revealed that they were intact. The results of histopathological and immunohistochemical findings are all shown in Figure 1, Figure 2, Figure 3 and Figure 4 and summarized in Table 3.

Three rats in the PNR group and two rats in the PNR + D3 group developed nodules classified as early neuromas, and no significant difference was detected between the two groups in terms of nodulation and foam cell proliferation (Figure 1a,b) (*p* > 0.05). No significant difference was also observed in collagen deposition between the PNR and the PNR + D3 groups (Figure 2a,b) (*p* > 0.05). The only significant change between the groups in histopathological examination was noted in Schwann cells, in that the PNR group had less increase in Schwann cells as compared to the PNR + D3 group, which showed a higher percentile of cells (Figure 3a,b) (*p* < 0.05). Although neurofilament immunoreactivity as a sign of axon regeneration was slightly lower in the PNR group compared to the PNR + D3 group, no statistically significant difference was detected between the groups (Figure 4a,b) (*p* > 0.05).

Between the NCI and the NCI + D3 groups, nodulation was only seen in one rat in the NCI group, and there was no significant difference between the groups (*p* > 0.05). No significant difference was also present in foam cell proliferation between the two groups (Figure 1c,d) (*p* > 0.05). However, collagen deposition was significantly higher in the NCI group, while there was no collagen deposition in the NCI + D3 group (Figure 2c,d) (*p* < 0.05). A higher percentile of increase in Schwann cells was also detected in the NCI group compared to the NCI + D3 group (Figure 3c,d) (*p* < 0.05). A higher percentile of axonal regeneration was detected in the NCI group compared to the NCI + D3 group (Figure 4c,d) (*p* < 0.05).

## 4. Discussion

The peripheral nervous system, having both somatic and autonomic functions, is a complicated system that enables numerous tasks. Damages to the peripheral nerves can cause a variety of functional losses depending on the severity and the type of injury [26]. Several approaches have been suggested on the effects of various medications on nerve healing to improve functional outcomes in peripheral nerve restoration [6,7,8,9,10]. In this study, we investigated the potential protective effect of intraperitoneal injection of vitamin D3 application in both transected and crushed peripheral nerve injuries.

Several studies have investigated the effect of vitamin D3 on neuronal degeneration. It has been indicated that daily intraperitoneal injection of 1 mcg/kg vitamin D3 in a diffuse axonal injury model in rats has protective effects against secondary axotomy after brain damage [27]. Effects of vitamin D in peripheral nerve repair have also been studied. In an experimental transected peripheral nerve model in rats, the use of 2.5 mcg/kg/day vitamin D2 for 10 weeks increased axogenesis and axon diameter, improved the response of sensory neurons, and induced a fast-to-slow fiber type transition, indicating major beneficial effects in peripheral nerve injury [22]. In another study, where the effects of vitamin D2 and vitamin D3 were compared in the transected nerve model, vitamin D3 provided better locomotor and electrophysiological recovery when given at 12.5 mcg/kg/day. The use of vitamin D3 resulted in a higher number of preserved and newly formed axons, a larger axon diameter, better myelination, and modified expression of genes involved in axogenesis and myelination [20]. Enhanced functional recovery and myelination were also observed with 5 mcg/kg/day vitamin D3 on facial nerve injury in rabbits [21]. In our investigation, the mean post-healing action potentials in all groups were significantly lower compared to those of the pre-healing counterparts. Although a slight increase in action potential was observed in the PNR + D3 group compared to the PNR group, the difference was not statistically significant. This slight increase in the mean action potential may suggest that more nerve fibers were repaired in vitamin D3-given nerve-transected animals. On the other hand, no significant improvement was observed in axonal regeneration in histopathological evaluation, yet a significant increase in Schwann cells was noted with vitamin D3 application in the rats with transected nerve, indicating that vitamin D3 may show some effect on the myelination process. Similar findings on the effects of vitamin D3 on myelination has also been previously reported [20]. Although significant change in the mean action potential value was not observed between the PNR and PNR + D3 groups, it was significantly decreased in the NCI + D3 group compared to the NCI group. This negative impact of vitamin D3 on the healing process of the crushed nerve after 12 weeks of healing period is an interesting finding. Furthermore, histological analysis of the crushed nerves revealed that vitamin D3 lowered the collagen synthesis and myelination during nerve healing while also decreasing the axon regeneration. These findings, both electrophysical and histopathological, indicate that vitamin D3 treatment may be more deleterious in crushed nerve injuries. Since previous research mostly focused on the transected injury, the effects of vitamin D3 in crushed injury may need more data for meaningful comparison.

We used the hot plate test to assess the acute pain and, hence, healing of the sensory nerve fibers. The test has been shown to be a simple and effective way of detecting central and peripheral hyperalgesia [28,29]. Post-healing reaction times were longer in the vitamin D3-treated groups as compared to the untreated counterpart groups, suggesting that vitamin D3 either inhibits sensory nerve repair or improves pain tolerance. Anti-inflammatory and immunomodulatory effects of vitamin D have been indicated, including possible analgesic properties [30]. Hypovitaminosis D has also been linked to chronic low back pain in female patients [31]. It has been suggested that intraperitoneal injection of 25 mcg/kg vitamin D3 supplementation reduces perceptions of cold allodynia and heat hyperalgesia in rats [32]. In the present investigation, although increased time frames were detected with vitamin D3 administration in the hot plate test, we were unable to differentiate whether the greater hot plate latency was caused by the negative effect of vitamin D3 on the sensory branches or its influence on the pain threshold, which needs to be investigated further.

The mechanism of crush injuries is distinct from that of full transections. A study of rat facial nerves found that NGF receptor mRNA levels were higher 3 to 14 days after damage in animals with crush injury compared to those with transection [33]. Functional recovery is substantially better after crush injuries than after nerve repair for transection injuries. In crush injuries, the emergence of axons in the distal stump occurs more quickly. It is hypothesized that in crush injuries, the endoneurial sheaths remain intact in the crushed area, providing paths for regenerated axons to go through the wounded nerve segment and reconnect with their prior peripheral connections [34]. The pathophysiology of peripheral nerve injury is not yet completely understood [35,36,37]. Therefore, the effect of injury type on the healing process cannot be explained by simple mechanisms. In our investigation, vitamin D3 appeared to have an adverse influence on the crush injury. Changes in the expression of neurotrophic factors or some yet unknown other modulatory proteins influence the healing, differentiating the processes in the transected and the crushed nerves. With the increased understanding of the pathophysiology of nerve injuries, these subjects may be clarified.

Depolarization time reflects the activation of voltage-dependent sodium (Na^+^) channels and represents early axonal excitability. In the present study, no significant differences were observed between pre-injury and post-healing depolarization times in the transection model, and vitamin D3 administration did not alter this parameter. Similarly, no differences were detected between the PNR and PNR + D3 groups, indicating that vitamin D3 did not significantly affect Na^+^ channel activation following transected nerve repair. These findings are consistent with previous observations that electrophysiological recovery after nerve transection is mainly influenced by axonal continuity and remyelination rather than early Na^+^ channel kinetics [4]. In contrast, the crush injury model demonstrated a distinct electrophysiological response. Vitamin D3–treated animals exhibited shorter post-healing depolarization times together with reduced action potential amplitudes, suggesting impaired conduction rather than enhanced excitability. Repolarization time, predominantly regulated by voltage-dependent potassium (K^+^) channels, was delayed after transection in both treated and untreated groups, consistent with impaired K^+^ channel function reported after nerve repair [4,38].

In crushed nerves, vitamin D3 treatment was associated with prolonged repolarization when compared with pre-injury values, indicating that the effects of vitamin D3 on depolarization and repolarization dynamics are injury-type dependent. It is known that when the membrane potential depolarizes to a threshold of roughly −45 mV, all voltage-dependent Na+ channels are activated, resulting in a fast depolarization phase [39]. The decreased conduction in the injured nerve segment in the crush injuries treated with vitamin D3 could be attributed to the lower activation of Na+ channels.

The contrasting results about the effects of vitamin D3 on the crush injury and transected injury may be explained by the differences in the regenerative mechanisms between the two injury types. It has been reported that Schwann cells play a significant role during the repair process in crush injuries by de-differentiation and proliferation, and inflammatory mechanisms contribute significantly to this process [40,41]. Since vitamin D has good immunomodulatory and anti-proliferative properties, it may suppress the regeneration of these cells early in the repair process [42]. Less regenerative activity of Schwann cells was observed in the vitamin D3-given crush injury group compared to the vitamin D3-given transected injury group in this study, which supports this notion.

In conclusion, we investigated the effect of vitamin D3 treatment on transected and cut injuries of peripheral nerve fibers. The results indicated that vitamin D3 has no significant positive effect on the transected nerve injury repair, yet it showed some degrees of beneficial outcomes on myelination and the action potential values, suggesting more nerve fiber regeneration. On the other hand, histopathological evaluations revealed a deleterious effect on the primary nerve healing with vitamin D3 treatment. Most strikingly, vitamin D3 treatment had a negative impact on the crushed nerve injury, which was assessed by electrophysiological measurements. Although vitamin D3 treatment was suggested to have beneficial effects on peripheral nerve injuries, the effects might be dose and time-dependent. In this investigation, acute vitamin D3 exposure was used, yet for the desired results, longer durations might be necessary.

## Figures and Tables

**Table 1:** Pre-injury vs. post-healing electrophysiological results across groups.

Parameters	Group	Pre-Injury	Post-Healing	*p* Value (Within-Group)
ΔPP (mV) mean ± SD	PNR	71.75 ± 4.13	19.38 ± 4.44	0.001
	PNR + D3	74.63 ± 5.80	26.71 ± 4.39	0.001
	*p* value (between-groups)	0.273	0.393	
	NCI	70.13 ± 5.41	48.38 ± 9.05	0.001
	NCI + D3	73.00 ± 4.14	38.88 ± 5.99	0.001
	*p* value (between-groups)	0.252	0.027	
ΔDT (ms) median (min–max)	PNR	0.85 (0.40–1.50)	1.00 (0.20–2.50)	0.327
	PNR + D3	0.68 (0.30–2.25)	1.00 (1.00–2.00)	0.175
	*p* value (between-groups)	0.399	0.948	
	NCI	0.70 (0.40–1.00)	1.00 (0.50–2.25)	0.173
	NCI + D3	0.70 (0.45–0.90)	0.45 (0.25–2.00)	0.482
	*p* value (between-groups)	0.120	0.032	
ΔRT (ms) median (min–max)	PNR	1.35 (0.90–2.50)	3.13 (1.50–4.80)	0.028
	PNR + D3	1.10 (0.70–3.00)	5.00 (1.50–7.50)	0.028
	*p* value (between-groups)	0.791	0.054	
	NCI	0.95 (0.70–3.70)	3.88 (1.25–5.00)	0.069
	NCI + D3	0.82 (0.60–2.00)	1.38 (0.60–5.00)	0.017
	*p* value (between-groups)	0.139	0.080	
ΔDT + ΔRT (ms) median (min–max)	PNR	1.98 (1.80–3.50)	5.00 (2.50–5.00)	0.025
	PNR + D3	2.75 (1.00–3.75)	6.00 (2.50–8.50)	0.028
	*p* value (between-groups)	0.845	0.050	
	NCI	1.90 (1.10–4.60)	4.75 (2.25–6.20)	0.036
	NCI + D3	1.35 (1.30–2.90)	1.88 (1.00–7.00)	0.208
	*p* value (between-groups)	0.055	0.027	

ΔPP is presented as mean ± SD. Within-group comparisons used a paired-samples *t*-test; between-groups comparisons used an independent-samples *t*-test. ΔDT, ΔRT, and ΔDT + ΔRT are presented as median (min–max). Within-group comparisons used the Wilcoxon signed-rank test; between-groups comparisons used the Mann–Whitney U test. Abbreviations: ΔPP, action potential amplitude; ΔDT, depolarization time; ΔRT, repolarization time; PNR, primary nerve repair; D3, vitamin D3 treatment; NCI, nerve crush injury; ms, milliseconds.

**Table 2:** Analysis of hot plate test parameters in the study groups.

Group	%MPE Median (Min–Max)
Control	7.00 (6.10–7.90) ^b^
PNR	6.90 (6.40–8.20) ^b^
PNR + D3	8.40 (7.10–9.10) ^a^
NCI	7.30 (6.10–8.20) ^b^
NCI + D3	8.00 (7.10–8.90) ^a^

Values are presented as median (min–max). Post hoc pairwise comparisons were performed using the Mann–Whitney U test; different letters within the column indicate statistically significant differences between groups (*p* < 0.05). Abbreviations: %MPE, percentage of maximum possible effect; PNR, primary nerve repair; D3, vitamin D3 treatment; NCI, nerve crush injury.

**Table 3:** Comparison of histopathological and immunohistochemical findings at 12 weeks after injury between groups with and without vitamin D3 treatment.

Histopathological and Immunohistochemical Findings	Category	PNR n (%)	PNR + D3 n (%)	*p* Value (PNR vs. PNR + D3) ^a^	NCI n (%)	NCI + D3 n (%)	*p* Value (NCI vs. NCI + D3) ^a^
Nodulation	No	5 (62.5)	6 (75.0)	0.590	7 (87.5)	8 (100.0)	0.302
	Yes	3 (37.5)	2 (25.0)		1 (12.5)	0 (0.0)	
Foam cells	No	1 (12.5)	1 (12.5)	0.999	8 (100.0)	7 (87.5)	0.302
	Yes	7 (87.5)	7 (87.5)		0 (0.0)	1 (12.5)	
Collagen deposition	No	3 (37.5)	1 (12.5)	0.248	4 (50.0)	8 (100.0)	0.021 *
	Yes	5 (62.5)	7 (87.5)		4 (50.0)	0 (0.0)	
Schwann cell increase	<%10	8 (100)	1 (12.5)	0.003 *	2 (25.0)	7 (87.5)	0.034 *
	%10–50	0 (0.0)	5 (62.5)		3 (37.5)	1 (12.5)	
	>%50	0 (0.0)	2 (25.0)		3 (37.5)	0 (0.0)	
Axon regeneration	<%10	5 (62.5)	2 (25.0)	0.131	1 (12.5)	0 (0.0)	0.022 *
	%10–50	0 (0.0)	0 (0.0)		0 (0.0)	5 (62.5)	
	>%50	3 (37.5)	6 (75.0)		7 (87.5)	3 (37.5)	

^a^ *p* values were calculated using the chi-square test. * *p* < 0.05 indicates statistical significance. Abbreviations: PNR, primary nerve repair; D3, vitamin D3 treatment; NCI, nerve crush injury.

## Data Availability

The data presented in this study are available on request from the corresponding author (the data are not publicly available due to privacy or ethical restrictions).

## Abbreviations

The following abbreviations are used in this manuscript:

PNR	Primary nerve repair
NCI	Nerve crush injury
MPE	Maximum probable effect
ΔPP	Action potential
ΔDT	Depolarization time
ΔRT	Repolarization time
∆DT + ∆RT	Total activation time
